# A Combined Effect of Expression Levels of Obesity-Related Genes and Clinical Factors on Cancer Survival Rate

**DOI:** 10.1155/2020/8838676

**Published:** 2020-11-24

**Authors:** Ting Huang, Xuan Huang, Yumin Nie, Xiangkui Shi, Chuanjun Shu

**Affiliations:** ^1^Department of Pharmacy, Xuzhou Maternity and Child Health Care Hospital, Xuzhou 221000, China; ^2^Reproductive Medical Center, Jinling Hospital Affiliated to The Medical School of Nanjing University, Nanjing 210002, China; ^3^Department of Bioinformatics, School of Biomedical Engineering and Informatics, Nanjing Medical University, Nanjing 211166, China

## Abstract

Obesity is directly associated with the risk of cancer in different organs, including breast, colon, and kidney. However, adipocytes could be utilized to control progression for some types of cancer, such as leukemia and breast cancer. To explore the potential correlation between adipocytes and cancer, the combined effect of expression levels of obesity-related genes and clinical factors (i.e., gender, race, menopausal status, history of smoking, tumor grade, body mass index (BMI), and history of drinking) on cancer survival rate was systemically studied. The expression levels of obesity-related genes in cancer tissues and normal tissues were downloaded from The Cancer Genome Atlas (TCGA). Kaplan–Meier curves were plotted using R programming language. The log-rank test was applied to explore the correlation between different clinical subgroups. The overexpression of the nine obesity-related genes (*MC4R*, *TMEM18*, *KCTD15*, *GNPDA2*, *SH2B1*, *MTCH2*, *FTO*, *PCSK1*, and *GPR120*) may associate with tumor-promoting factors in some organs (head and neck, gastrointestinal tract, liver, and gallbladder). Underexpressed *LEPR*, *NEGR1*, *TMEM18*, and *SH2B1* genes prevented the progression and metastasis of kidney cancer. The combined effect of clinical factors and the expression levels of obesity-related genes on patients' survival was found to be significant. Our outcomes suggested that the alternations of DNA methylation patterns could result in the changes of expression levels of obesity-related genes, playing a critical role in tumor progression. The results of the current study may be utilized to supplement precision and personalized medicine, as well as provide novel insights for the development of treatment approaches for cancer.

## 1. Introduction

Cancer is a group of diseases involving the uncontrollable growth of abnormal cells with the potential to invade or spread to the other parts of the body [1]. In the 21^st^ century, cancer is the leading cause of human deaths, as well as the foremost barrier to extended life expectancy worldwide [2]. The global incidence and mortality of cancer are rapidly increasing due to the growth and aging of the population, as well as changes in the prevalence and distribution of the main risk factors for cancer [3–5]. Right now 24.6 million people are living with cancer, and by 2020, it is projected that there will be 16 million new cancer cases and 10 million cancer deaths every year (http://www.who.int). For China, the crude cancer incidence rate (CCIR) was 278.07/100,000 [6]. When considering cancer types, lung (CCIR = 57.13/100,000), breast (CCIR = 41.82/100,000),stomach (CCIR = 30.00/100,000), colorectum (CCIR = 27.08/100,000), and liver cancer (CCIR = 26.67/100,000) were the most common five cancers in whole Chinese population [6].

Obesity is associated with several types of cancer [7–10]. The prevalence of obesity has substantially increased worldwide [11]. At present, adult obesity rates in the United States reached 36.2%, with 67.9% being overweight, whereas in 1975, obesity did not exceed 11.9% [12]. Dramatically, in the pediatric and adolescent population, the prevalence rate of obesity reached 21.4% [12–14]. The increasing obesity state is generally caused by a lack of physical activity, unhealthy eating patterns resulting in excess energy intake, or a combination of the two resulting in energy excess [15]. Adipocytes and their functionally related cells are strong candidates for the promotion of carcinogenesis, as well as influencing the tumor microenvironment [16]. A previous study indicated that dietary lipids may promote the metastasis of cancer cells [8]. Adipocyte-ovarian cancer cell coculture led to the direct transfer of lipids from adipocytes to ovarian cancer cells and promoted in vitro and in vivo tumor growth [17]. Moreover, adipocytes may act as an energy source for the cancer cells [7, 17]. The above-mentioned results indicated that adipocytes may play a critical role in the carcinogenic process for several types of cancer.

However, boosting adipocytes or fat cells located in the bone marrow not only suppresses cancerous leukemia cells but also induces the regeneration of red blood cells [18]. This result suggested that adipocytes play a critical role in the tumor microenvironment. Another study revealed that high body mass index (BMI) was positively associated with the incidence of several types of cancer, while patients with high BMI at the time of initial diagnosis had higher two/five-year survival rates than those with low BMI [4]. These outcomes demonstrated that adipocytes may play a significant role in the suppression process of some types of cancer cells. Therefore, the correlation between adipocytes and cancer needs to be further explored.

The effects of obesity-related genes on cancer survival rate were investigated to deduce the role of adipocytes in solid tumors, since obesity-related genes play a pivotal role in the accumulation and distribution of adipocytes [19]. In the present study, 13 well-known obesity-related genes, including leptin receptor (*LEPR*), proopiomelanocortin (*POMC*), melanocortin 4 receptor (*MC4R*), transmembrane protein 18 (*TMEM18*), potassium channel tetramerization domain containing 15 (*KCTD15*), glucosamine-6-phosphate deaminase 2 (*GNPDA2*), SH2B adapter protein 1 (*SH2B1*), mitochondrial carrier homolog 2 (MTCH2), neuronal growth regulator 1 (*NEGR1*), fat mass and obesity-associated protein (*FTO*), leptin (*LEP*), proprotein convertase 1 (*PCSK1*), and G-protein coupled receptor 120 (*GPR120*) [20–23], were studied. In addition, the combined effect of expression levels of obesity-related genes and clinical factors on cancer survival rate was assessed.

## 2. Materials and Methods

### 2.1. Data Preparation

To improve the diagnosis, treatment, and prevention of cancer, a project supervised by the National Cancer Institute's Center for Cancer Genomics and the National Human Genome Research Institute (Bethesda, MD, USA), namely, The Cancer Genome Atlas (TCGA), was commenced in 2015 [24, 25]. According to TCGA database, RNA-seq data of 33 types of cancer were downloaded. These RNA-seq data were obtained from 8138 cancer tissues and their corresponding 737 normal tissues. Then, a file that included the gene expression levels was retrieved using TCGA assembler [26, 27]. According to a previous study [26], the estimation of transcripts was multiplied by 10^6^ for obtaining transcripts per million (TPM).

The clinical data of each type of cancer were downloaded using Genomic Data Commons (GDC) Data Transfer Tool (https://gdc.cancer.gov/) [28], encompassing 9651 cancer patients. The clinical data included patients' age, gender, overall survival, height and weight, history of smoking, history of drinking, race, menopausal status, and tumor grade. Notably, the race-based data of 8599 cancer patients were available, history of smoking records of 1332 cancer patients could be obtained, the height- and weight-based data of 2435 cancer patients were accessible, and menopausal status records of 1439 cancer patients could be downloaded.

### 2.2. Data Analysis

Cancer patients were divided into different subgroups based on clinical factors, including gender (male or female), race (African-American, Caucasian, and Asian), menopausal status (premenopause, perimenopause, and postmenopause), history of smoking (smoker, nonsmoker, reformed smoker #1 (≤15 years), and reformed smoker #2 (>15 years)), tumor grade (grade 1, grade 2, grade 3, and grade 4), body weight (normal weight, extreme weight, obese, and extreme obese), and history of drinking (occasional drinker, social drinker, daily drinker, weekly drinker, and nondrinker) [4]. Patients in male- and female-specific types of cancer were not taken into consideration when the combined effect of gender and expression levels of obesity-related genes on the cancer survival rate was explored.

The patients were divided into four groups based on BMI: normal weight (18 kg/m^2^ ≤ BMI < 25 kg/m^2^), high weight (25 kg/m^2^ ≤ BMI < 30 kg/m^2^), obese (30 kg/m^2^ ≤ BMI<40 kg/m^2^), and extremely obese (BMI ≥ 40 kg/m^2^). The Student *t*-test was used to estimate the significance of difference in the expression levels of obesity-related genes between different subgroups [29]. Among the top 25 over/underexpressed genes, genes that had significantly different TPMs were selected and then sorted based on the following equation: (mean TPM in cancer tissues)/(mean TPM in normal tissues).

The expression levels of obesity-related genes in cancer tissues and normal tissues were compared to elucidate the role of obesity-related genes in the incidence of cancer. Since the expression levels of obesity-related genes could be influenced by clinical factors, Kaplan–Meier analysis was used to explore the combined effect of expression levels of obesity-related genes and clinical factors on the cancer survival rate. According to TPM values, samples were firstly divided into two groups. When the TPM value was above the upper quartile, patients were assigned to the high-expression level group, while those with TPM value below the upper quartile were assigned to the low/medium-expression level group. Then, patients in each group were further divided into subgroups based on the clinical factors. Kaplan–Meier curve was plotted using the R programming language [30]. The log-rank test was utilized to calculate the *P* value to indicate the correlation between different groups [31]. The expression levels of obesity-related genes in each type of cancer were analyzed by multivariate regression analysis.

Furthermore, alteration of DNA methylation patterns is a hallmark of cancer. Moreover, DNA methylation regulates gene expression. Hence, DNA methylations of obesity-related genes for each type of cancer were downloaded from TCGA. The *β* values for each probe were calculated using the formula provided by Illumina: *β* = *M*/(*U* + *M* + 100), where *M* is methylated intensity and *U* denotes unmethylated intensity. The *β* values were set from 0 (unmethylated) to 1 (fully methylated). Mutations, copy number variations (CNVs), and controlling expression levels of obesity-related genes were downloaded from TCGA.

## 3. Results

### 3.1. Expression Levels of Obesity-Related Genes in Cancer Tissues and Normal Tissues

As mentioned earlier, the RNA-seq data of 8138 cancer tissues and their corresponding 737 normal tissues were downloaded from TCGA database. The cancer tissues consisted of 21 types of cancer, including bladder urothelial carcinoma (BLCA), breast invasive carcinoma (BRCA), cervical squamous cell carcinoma (CESC), cholangiocarcinoma (CHOL), colon adenocarcinoma (COAD), esophageal carcinoma (ESCA), glioblastoma multiforme (GBM), head and neck squamous cell carcinoma (HNSCC), kidney chromophobe (KICH), kidney renal clear cell carcinoma (KIRC), kidney renal papillary renal cell carcinoma (KIRP), liver hepatocellular carcinoma (LIHC), lung adenocarcinoma (LUAD), lung squamous cell carcinoma (LSCC), pancreatic adenocarcinoma (PAAD), pheochromocytoma/paraganglioma (PCPG), prostate adenocarcinoma (PRAD), rectum adenocarcinoma (READ), stomach adenocarcinoma (STAD), thyroid carcinoma (THCA), and uterine corpus endometrial carcinoma (UCEC). To assess the effects of expression levels of obesity-related genes on cancer survival rate, the expression levels of the 13 obesity-related genes (*LEPR*, *POMC*, *MC4R*, *TMEM18*, *KCTD15*, *GNPDA2*, *SH2B1*, *MTCH2*, *NEGR1*, *FTO*, *LEP*, *PCSK1*, and *GPR120*) were compared between normal tissues and cancer tissues.

**Figure 1:**
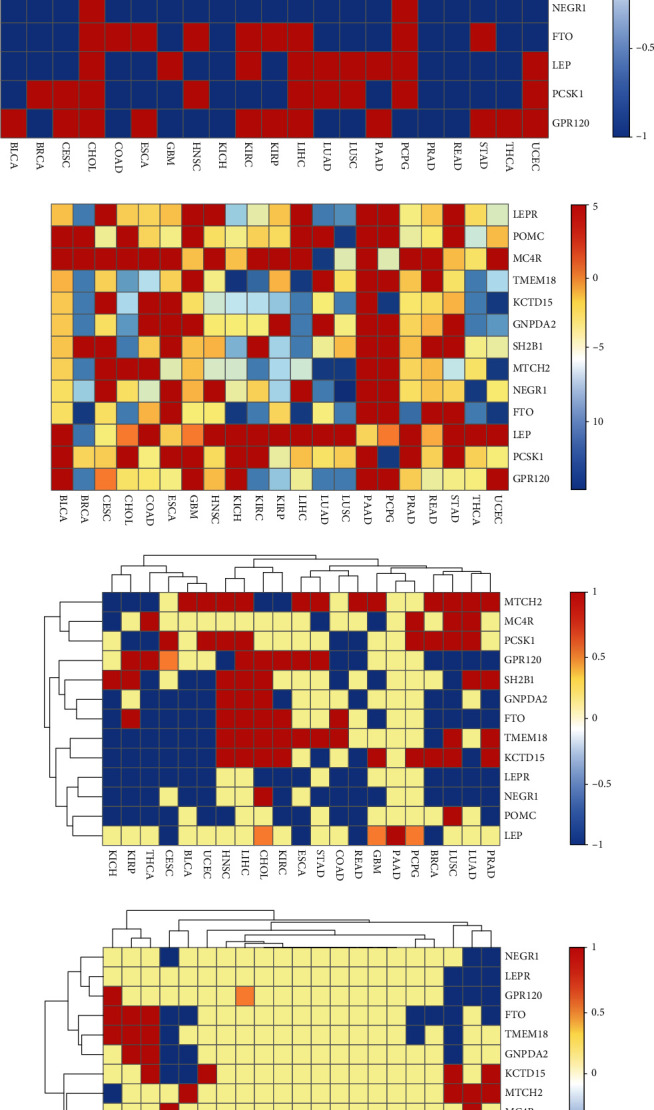
Differences in expression levels of obesity-related genes between cancer tissues and normal tissues. (a) Comparing the expression levels of obesity-related genes in different tissues for different types of cancer. Blue (denoted as -1) and red (denoted as 1) colors represent that the expression levels of the obesity-related genes in cancer tissues were higher and lower than those in the normal tissues, respectively. (b) *P* value was plotted in a log10 scale. The red (denoted as 5) indicates insignificant difference in expression levels of obesity-related genes between cancer tissues and normal tissues. Other colors represent the *P* value in a log10 scale. (c) Integration of (a) and (b). Comparing the expression levels of obesity-related genes in normal tissues, red (value = 1) and blue (value = −1) indicated significant upregulation and downregulation of expression levels of obesity-related genes in cancer tissues. Moreover, yellow (value = 0) and brown (value = 0.5) colors mean the existence of insignificant difference between expression levels of obesity-related genes in different tissues. (d) The same as (c) when *P* value was set to ≤1*E*-10.

As shown in Figure 1(a), the expression levels of four obesity-related genes (*LEPR*, *POMC*, *MC4R*, and *NEGR1*) in almost all types of cancer tissues were lower than those in the corresponding normal tissues. Conversely, the expression levels of LEPR, POMC, MC4R, and NEGR1 in two (GBM and PAAD), two (CHOL and LUSC), four (LUAD, LUSC, PCPG, and THCA), and two (CHOL and PCPG) types of cancer were higher than those of the corresponding normal tissues, respectively. However, the expression level of one obesity-related gene, *MTCH2*, in the majority of types of cancer tissues was higher than that in the corresponding normal tissues. Conversely, expression level of *MTCH2* in seven (CHOL, COAD, KICH, KIRC, KIRP, PAAD, and THCA) types of cancer was lower than that in the corresponding normal tissues. The expression levels of other eight obesity-related genes (*TMEM18*, *KCTD15*, *GNPDA2*, *SH2B1*, *FTO*, *LEP*, *PCSK1*, and *GPR120*) in about half types of cancer tissues were higher/lower than those in the corresponding normal tissues.

However, no significant difference was detected in the expression levels of obesity-related genes among cancer tissues and normal tissues (*P* > 0.05, Figure 1(b)). For example, the difference in expression levels of each of the 13 obesity-related genes between PAAD and PCPG types of cancer was not statistically significant, and the difference in the expression levels of *LEP* and *MC4R* in the 21 types of cancer was not significant. The statistical difference in the expression levels of obesity-related genes between cancer tissues and normal tissues was analyzed and illustrated in Figure 1(c) (red: high; blue: low). Compared to the normal tissues, the expression level of each of the three obesity-related genes (*LEPR*, *NEGR1*, and *POMC*) in cancer tissues was found to be insignificant. The expression level of each of MC4R and LEP in several types of cancer tissues did not alter significantly. The expression level of MTCH2 in various types of cancer tissues increased. The expression level of each of the five obesity-related genes (*SH2B1*, *GNPDA2*, *FTO*, *TMEM18*, and *KCTD15*) in three cancer tissues (HNSC, LIHC, and CHOL) was higher than that in the corresponding normal tissues. Notably, the expression levels of the 13 obesity-related genes in PAAD between cancer tissues and the corresponding normal tissues were not statistically significant, except for LEP. When *P* value was <1*E*-10, the difference in the expression level of an obesity-related gene between cancer and normal tissues was statistically significant, and the gene was defined as a “significant obesity-related gene.” As illustrated in Figure 1(d), the 13 obesity-related genes in 11 types of cancer (KIRC, CHOL, LIHC, THCA, UCEC, PCPG, KICH, PRAD, BRCA, LUAD, and LUSC) were considered as significant obesity-related genes.

### 3.2. Effects of Expression Levels of Obesity-Related Genes on Cancer Survival Rate

**Figure 2:**
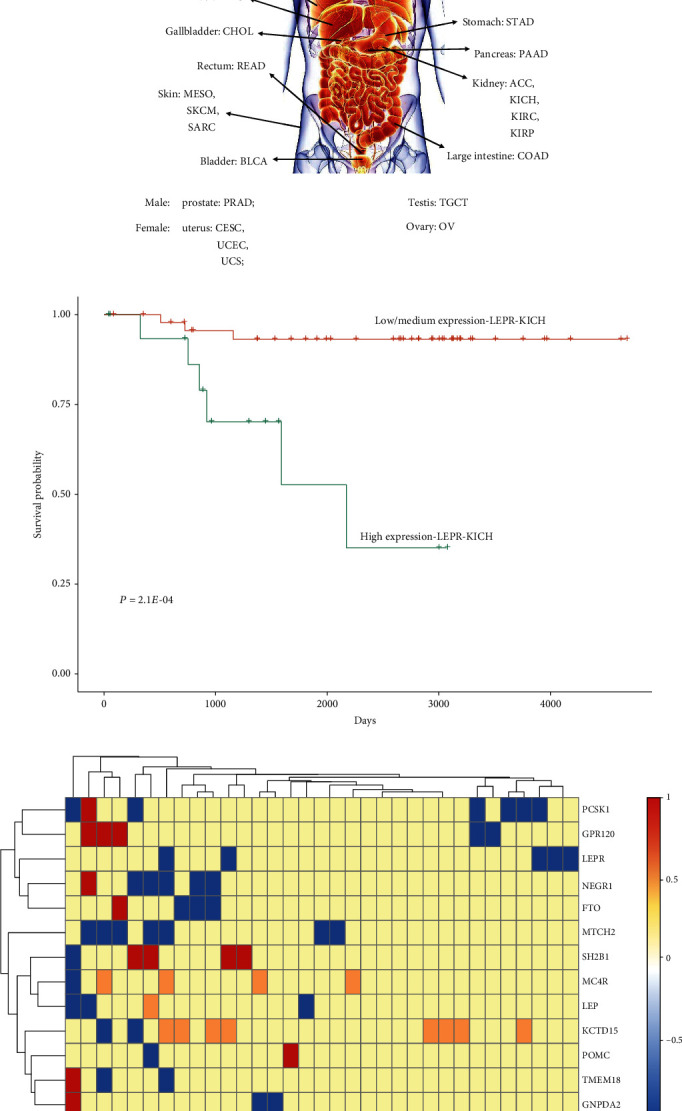
Effects of expression levels of obesity-related genes on cancer survival rate. (a) Distribution of different types of cancer in tissues. (b) The effect of expression level of *LEPR* gene on patients' survival with KICH. Days represent the survival time after diagnosis. Red and blue lines indicate low/medium expression level (with TPM values below the upper quartile) and high-expression level groups (with TPM values above the upper quartile) for *LEPR* gene in LIHC patients, respectively. (c) This shows the effects of expression levels of the 13 obesity-related genes on cancer survival rate for each of the 33 types of cancer. Red (value = 1)/blue (value = −1) represents that patients in high-expression level group have a higher/lower survival probability than those in low/medium-expression group. Yellow (value = 0) and brown (value = 0.5) colors indicate the insignificant difference in the obesity-related gene between high- and low/medium-expression level groups.

The expression levels of obesity-related genes were categorized into two groups: high-expression level group (with TPM values above the upper quartile) and low/medium-expression level group (with TPM values below the upper quartile). The cohort consisted of 9651 cancer patients with accessible expression levels, of whom 2502 cancer patients were in the high-expression level group and 7149 cases were in the low/medium-expression level group. The types of cancer not only included the above-mentioned 21 types of cancer but also involved an additional 12 types of cancer (adrenocortical carcinoma (ACC), low-grade glioma (LGG), acute myeloid leukemia (AML), diffuse large B cell lymphoma (DLBCL), mesothelioma (MESO), ovarian serous cystadenocarcinoma (OSC), sarcoma (SARC), skin cutaneous melanoma (SKCM), testicular germ cell tumor (TGCT), thymoma (THYM), uterine carcinosarcoma (UCS), and uveal melanoma (UVM)) (Figure 2(a)). To investigate the effects of expression levels of obesity-related genes on cancer survival rate, Kaplan–Meier curve was plotted to estimate the cancer survival rate in different expression level-based groups for each of the 33 types of cancer (Figure 2(b)).

Kaplan–Meier analysis was utilized to plot the overall survival curve. For instance, as shown in Figure 2(b), Kaplan–Meier survival plots for each of the expression level-based groups for *LEPR* gene of KICH patients were plotted. According to the Kaplan–Meier survival curves, the cancer survival rate in the *LEPR* gene between high- and low/medium-expression level groups in KICH patients was statistically significant (*P* = 2.10*E* − 04). This result indicated that KICH patients with a high expression level of the *LEPR* gene might have a low cancer survival rate. Also, the cancer survival rate between the two expression level-based groups for each of the 13 obesity-related genes in each of the 33 types of cancer was calculated. As displayed in Figure 2(c), for each of the five obesity-related genes (*LEPR*, *MTCH2*, *MC4R*, *LEP*, and *KCTD15*), the patients in a high-expression group had a greater cancer survival rate than those in the low/medium-expression groups (*P* < 0.05). For instance, for each of the 5 types of cancer (KICH, CESC, BLCA, HNSC, and SARC), patients in the low/medium-*LEPR* expression group had higher cancer survival rate than those in the high-*LEPR* expression group (*P* < 0.05). However, for the other eight obesity-related genes, the correlation between expression level and cancer survival rate was complicated. For patients with six types of cancer (KIRC, LUAD, LGG, GBM, UCEC, and BLCA), those in the *PCSK1* low/medium-expression group had higher survival probability than those in the high-expression group. However, for patients with SKCM, patients with high expression level of *PCSK1* may benefit from a superior survival probability than those with low/medium expression level. Furthermore, for the 16 types of cancer (KICH, OV, STAD, THCA, LIHC, THYM, COAD, BRCA, MESO, LGG, ESCA, GBM, UCEC, BLCA, HNSC, and SARC), patients in the high-expression level group did not benefit from higher survival probability as compared to patients in the low/medium-expression level groups. However, for LUSC and PAAD, patients with low/medium expression levels of obesity-related genes could not attain a higher survival probability as compared to patients with high expression levels.

### 3.3. Methylation, Mutations, and CNVs of Obesity-Related Genes for Solid Tumors

**Figure 3:**
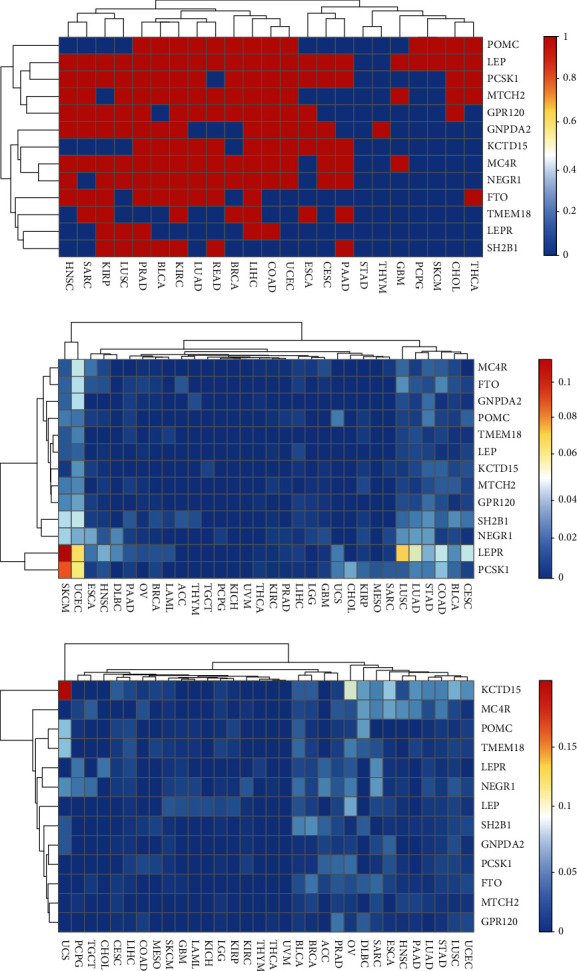
The alterations of methylation, mutations, and CNVs for obesity-related genes in solid tumors. (a) DNA methylation patterns of obesity-related genes. DNA methylation levels of cancer tissues and normal tissues were compared to indicate the alteration of DNA methylation patterns. (b) Mutation rates of obesity-related genes in solid tumors. The colors represent the corresponding mutation rates. (c) The CNV rates of obesity-related genes in solid tumors.

DNA methylation is a major epigenetic modification that is strongly involved in the physiological control of genome expression. The DNA methylation patterns have been extensively improved in cancer cells and, therefore, can be used to distinguish cancer cells from normal tissues. The alteration of DNA methylation patterns is a hallmark of cancer. Then, the *β* values of obesity-related genes for cancer tissues and normal tissues were compared to indicate the alteration of DNA methylation patterns (Figure 3(a); red represents difference and blue denotes identity). According to the results of cluster analysis, the levels of DNA methylation for obesity-related genes in almost all types of cancer (HNSC, SARC, KIRP, LUSC, PRAD, BLCA, KIRC, LUAD, READ, BRCA, LIHC, and COAD) have changed (Figure 3(a)). Compared to the normal tissues, the five obesity-related genes (POMC, LEP, PCSK1, MTCH2, and GPR120) showed a similarity alteration of DNA methylation patterns in different cancer tissues (Figure 3(a)).

The CNV has gained attention as a type of genomic/genetic variation that plays a pivotal role in disease susceptibility. CNV is consisted of fusion, amplification, and deep deletion. The mutation and CNV rates of each one of the 13 obesity-related genes in solid tumors were calculated (Figure 3). As shown in Figure 3(b), the mutation rates of most obesity-related genes in cancer tissues were <0.05. However, the mutation rates of five obesity-related genes in few cancer tissues were >0.05 (i.e., *LEPR* in SKCM (0.11), UCEC (0.07), and LUSC (0.07) and *PCSK1* in SKCM (0.09) and UCEC (0.06)). The CNV rates of obesity-related genes in cancer tissues were also <0.05. As depicted in Figure 3(c), the CNV rates of five obesity-related genes in cancer tissues were >0.05 (i.e., *KCTD15* in ESCA (0.06), OSC (0.08), and UCS (0.19); *POMC* in UCS (0.06); *TMEM18* in UCS (0.06)). These findings indicated that the mutation and CNV rates of obesity-related genes may not play a remarkable role in carcinogenic process of most solid tumors, except for DNA methylation patterns.

### 3.4. Combined Effect of Clinical Factors and Expression Levels of Obesity-Related Genes on Cancer Survival Rate

In order to explore the combined effect of clinical factors and expression levels of obesity-related genes on the cancer survival rate, samples were categorized using seven clinical factors, including patients' gender, race, menopausal status, history of smoking, tumor grade, BMI, and history of drinking. Then, for each of the 33 types of cancer, the expression levels of the 13 obesity-related genes were divided into different groups based on the seven factors as follows: (1) patients' gender (male or female), (2) patients' race (African-American, Caucasian, and Asian), (3) menopausal status (premenopause, perimenopause, and postmenopause), (4) history of smoking (smoker, nonsmoker, reformed smoker #1 (≤15 years), and reformed smoker #2 (>15 years)), (5) tumor grade (grade 1, grade 2, grade 3, and grade 4), (6) BMI (normal weight (18 kg/m^2^ ≤ BMI < 25 kg/m^2^), high weight (25 kg/m^2^ ≤ BMI < 30 kg/m^2^), obese (30 kg/m^2^ ≤ BMI < 40 kg/m^2^), and extremely obese (BMI ≥ 40 kg/m^2^)), and (7) history of drinking (occasional drinker, social drinker, daily drinker, weekly drinker, and nondrinker).

**Figure 4:**
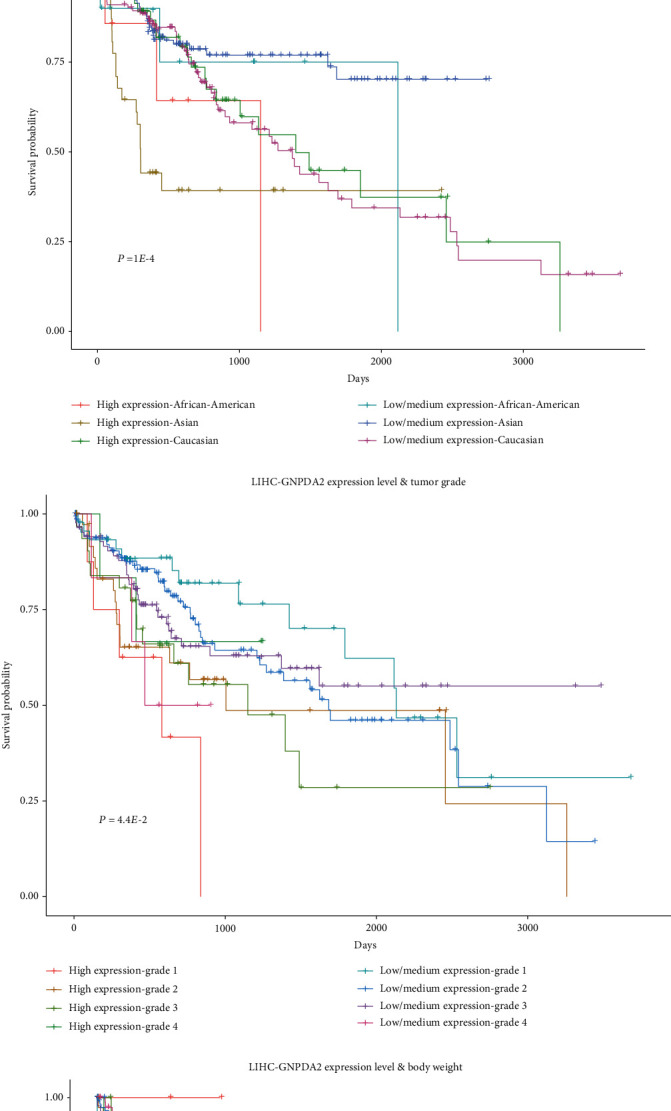
Kaplan–Meier survival curves for LIHC patients with different expression levels of *GNPDA2* along with different genders, races, tumor grades, and body weight. (a) The combined effect of expression level of *GNPDA2* and gender on the survival probability of LIHC patients. The patients were assigned to four groups: high-expression level female, high-expression level male, low-expression level female, and low-expression level male. (b) The combined effect of the expression level of *GNPDA2* and race on the survival probability of LIHC patients. The patients were divided into six groups: high-expression level African-American, high-expression level Asian, high-expression level Caucasian, low-expression level African-American, low-expression level Asian, and low-expression level Caucasian. (c) The combined effect of expression level of *GNPDA2* and tumor grade on the survival probability of LIHC patients. The patients were divided into eight groups: high-expression level grade 1, high-expression level grade 2, high-expression level grade 3, high-expression level grade 4, low-expression level grade 1, low-expression level grade 2, low-expression level grade 3, and low-expression level grade 4. (d) The combined effect of expression level of *GNPDA2* and BMI on the survival probability of LIHC patients. The patients were divided into eight groups: high expression-normal weight (18 kg/m^2^ ≤ BMI < 25 kg/m^2^), high expression-high weight (25 kg/m^2^ ≤ BMI < 30 kg/m^2^), high expression-obese (30 kg/m^2^ ≤ BMI < 40 kg/m^2^), high expression-extremely obese (BMI ≥ 40 kg/m^2^), low expression-normal weight (18 kg/m^2^ ≤ BMI < 25 kg/m^2^), low expression-high weight (25 kg/m^2^ ≤ BMI < 30 kg/m^2^), low expression-obese (30 kg/m^2^ ≤ BMI < 40 kg/m^2^), and low expression-extremely obese (BMI ≥ 40 kg/m^2^).

In the current study, Kaplan–Meier survival curves for each of the obesity-related genes for the same type of cancer were compared using the SPSS 19.0 software (IBM, Armonk, NY, USA). For instance, for *GNPDA2* gene in LIHC patients, four clinical factors (gender (Figure 4(a), *P* = 1.1*E* − 3), race (Figure 4(b), *P* = 1*E* − 4), tumor grade (Figure 4(c), *P* = 4.4*E* − 2), and BMI (Figure 4(d), *P* = 2.1*E* − 2)) influenced the Kaplan–Meier survival curves in cancer tissues with different expression levels of the *GNPDA2* gene. Furthermore, male patients with different expression levels of the *GNPDA2* gene were found (*P* < 1*E* − 4) (Figure 4(a)). However, the difference was not statistically significant (*P* = 0.88) for female patients. This result further indicated that gender along with altered expression levels of *GNPDA2* may affect Kaplan–Meier survival curves of LIHC patients. For the other three clinical factors, i.e., menopausal status, history of smoking, and history of drinking habit, there were no data in TCGA database for LIHC patients.

**Figure 5:**
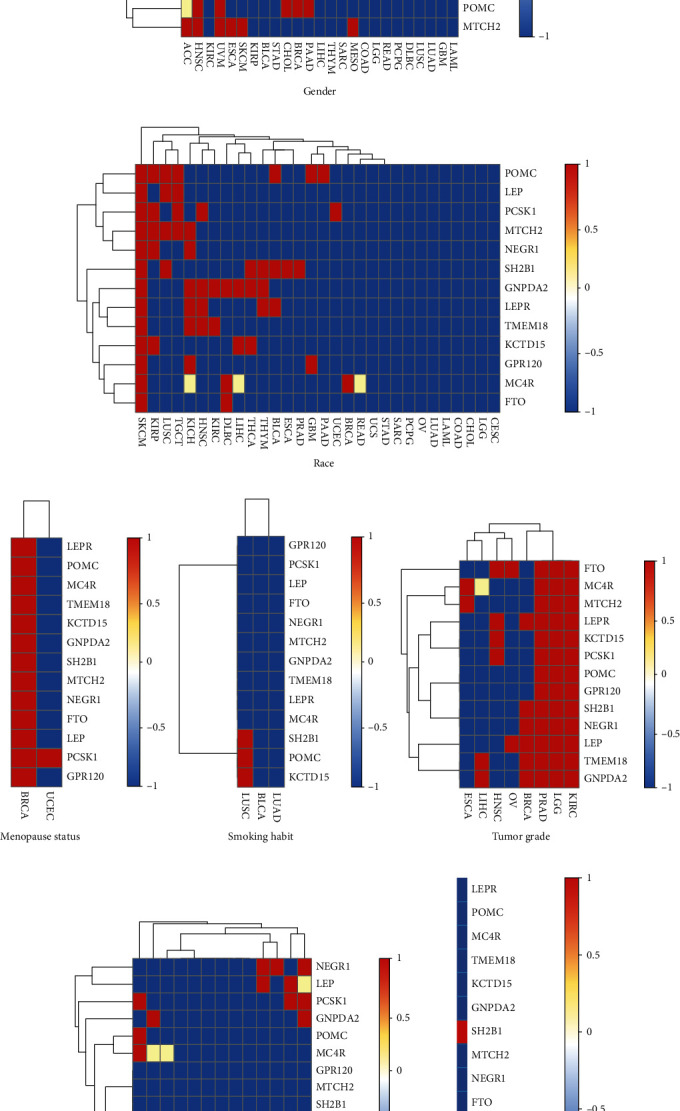
Combined effect of clinical factors and expression levels of obesity-related genes on cancer survival rate. Red (value = 1), blue (value = −1), and yellow (value = 0.5) represent the remarkable effect, meaningless effect, and unknown, respectively. (a–g) Indicate the combined effects of gender and expression level, race and expression level, menopausal status and expression level, history of smoking and expression level, tumor grade and expression level, BMI and expression level, and history of drinking and expression level, respectively.

In clinical data of 33 types of cancer, gender-based records of 25 types of cancer were available (except for CESC, KICH, OV, PRAD, TGCT, THCA, UCS, and UCEC); race-based data of 30 types of cancer were recorded in TCGA (except for ACC, MESO, and UVM); menopausal status-based records were only present in two types of cancer (BRCA and UCEC); history of smoking-based data of three types of cancer (LUSC, BLCA, and LUAD) were accessible; tumor grade-based data of eight types of cancer (ESCA, LIHC, HNSC, OV, BRCA, PRAD, LGG, and KIRC) were recorded; BMI-based records of cancer patients were available in 13 types of cancer (CHOL, READ, UCEC, THYM, SKCM, DLBC, KIRP, COAD, ESCA, LIHC, UVM, BLCA, and CESC); history of drinking-based data in one type of cancer could be obtained. The combined effect of clinical factors and expression levels of obesity-related genes on Kaplan–Meier survival curves of each type of cancer was calculated, and then, the significant effect was highlighted in Figure 5 by red color. As shown in Figure 5(a), the gender and expression levels of six obesity-related genes (GNPDA2, KCTD15, LEPR, SH2B, POMC, and MTCH2) could remarkably affect the survival probability of patients with 14 types of cancer (ACC, HNSC, KIRC, UVM, ESCA, SKCM, KIRP, CHOL, BRCA, PAAD, LIHC, THYM, SARC, and MESO). Notably, the gender and expression levels of six obesity-related genes influenced the survival probability of patients with HNSC. However, the survival probability of the other types of cancer in these 13 types of cancer was influenced by gender and expression levels of one/two obesity-related genes. In addition, gender and expression levels of other seven obesity-related genes (*GPR120*, *LEP*, *MC4R*, *TMEM18*, *PCSK1*, *NEGR1*, and *FTO*) could markedly affect survival probability of patients with 11 types of cancer (ACC, KIRC, UVM, ESCA, SKCM, KIRP, BLCA, STAD, CHOL, COAD, and LGG). Among these, only survival probability of KIRC patients could be impacted by gender and expression levels of four obesity-elated genes (*LEP*, *MC4R*, *TMEM18*, and *PCSK1*). The remaining of these 11 types of cancer were only influenced by gender and expressions levels of <3 obesity-related genes.

Race and expression levels of five obesity-related genes (*POMC*, *LEP*, *PCSK1*, *MTCH2*, and *NEGR1*) could remarkably impact the survival probability of patients with ten types of cancer (SKCM, KIRP, LUSC, TGCT, KICH, HNSC, BLCA, GBM, PAAD, and UCEC), especially SKCM, KIRP, LUSC, and TGCT (Figure 5(b)). However, race and expression levels of the other eight obesity-related genes (*SH2B1*, *GNPDA2*, *LEPR*, *TMEM18*, *KCTD15*, *GPR120*, *MC4R*, and *FTO*) noticeably affected the survival probability of patients with 15 types of cancer (SKCM, KIRP, LUSC, KICH, HNSC, KIRC, DLBC, LIHC, THCA, THYM, BLCA, ESCA, PRAD, GBM, and BRCA), especially SKCM and KICH.

Menopausal status and expression levels of all obesity-related genes could markedly influence the survival probability of BRCA patients (Figure 5(c)). However, menopausal status and expression level of PCSK1 affected the survival probability of UCEC patients. For three types of cancer (LUSC, BLCA, and LUAD), history of smoking and expression levels of three obesity-related genes (*SH2B1*, *POMC*, and *KCTD15*) influenced the survival probability of patients with LUSC (Figure 5(d)). For history of smoking and expression levels of other obesity-related genes, no significant difference was detected among different groups in terms of survival probability for each of the three types of cancer. As illustrated in Figure 5(f), tumor grade and expression levels of obesity-related genes (*PRAD*, *LGG*, and *KIRC*) affected the survival probability of patients with all types of cancer. BMI and expression levels of obesity-related genes could influence the survival probability of patients with several types of cancer, such as CHOL, LIHC, BLCA, CESC, UCEC, and UVM (Figure 5(f)). As shown in Figure 5(g), history of drinking and expression levels of obesity-related genes also impact the survival probability of patients with PAAD.

### 3.5. Distribution of Obesity Genes and Their Related Genes in Top 25 over/Underexpressed Genes

Over/underexpressed genes are utilized to explore their roles in the occurrence and development of tumors. Hence, obesity genes in this study and their related genes were searched in the top 25 over/underexpressed genes to indicate whether they may play significant roles in the occurrence and development of tumors. To identify the related genes, the corresponding proteins of the obesity genes were identified (Table 1). To obtain the top 25 over/underexpressed genes, Comprehensive Perl Archive Network (CPAN) module, namely, “Statistics::Descriptive,” was employed to obtain the mean TPM value of each gene in cancer tissues and normal tissues, respectively. These top 25 over/underexpressed genes were selected and sorted for every type of cancer (Supplementary Table S1) according to the following formula: (mean TPM in cancer tissues)/(mean TPM in normal tissues). Then, the obesity genes and their related genes were compared to the top 25 over/underexpressed genes for each type of cancer (Table 1).

A total of eight obesity genes or their related genes were distributed among the top 25 over/underexpressed genes in some types of cancer (Table 1, supplementary Table S2). As shown in Table 1, one obesity gene (*LEP*, underexpressed gene 1) and one related gene (*ADIPOQ*, underexpressed gene 9) were distributed in the top 25 over/underexpressed genes for BRCA patients; 2 related genes (underexpressed gene 18: GCG; underexpressed gene 3: *PYY*) were included in the top 25 over/underexpressed genes for COAD patients; one related gene was distributed in the top 25 over/underexpressed genes for each of THCA (overexpressed gene 21: *PCSK1N*) and UCEC (overexpressed gene 2: *TFAP2A*) patients; one obesity gene was found in the top 25 over/underexpressed genes for *BLCA* (underexpressed gene 11: *NEGR1*) patients. In addition, five related genes of obesity (*ADIPOQ*, *GCG*, *PCSK1N*, *TFAP2A*, and *PYY*) were distributed in the top 25 over/underexpressed genes of cancer tissues.

## 4. Discussion

In the present study, the combined effect of obesity-related genes on cancer survival rate was systemically investigated. The expression levels of obesity-related genes between cancer tissues and normal tissues were explored to elucidate the association between the occurrence of cancer and expression levels of obesity-related genes. Leptin is an important regulator of adipose tissue mass and has been associated with tumor cell growth [32]. Meanwhile, LEP is an adipocyte-specific hormone that regulates body weight through hypothalamus effects [20]. Furthermore, leptin may modify estrogenic activity by inducing aromatase activity, thereby increasing the amount of androstenedione converted to estrone in adipose tissue [33]. The *POMC* gene has been identified as a major target of leptin and insulin action [34]. NEGR1 is a raft-associated extracellular protein that participates in cell recognition and interaction, which is crucial for control of growth and malignant transformation [35].

The molecular and cellular functions of the nine obesity-related genes were not fully understood. For instance, *GPR120* functions as a receptor for unsaturated long-chain free fatty acids and plays a significant role in sensing dietary requirement and in regulating the energy balance [36]. However, GPR120 induces angiogenesis and migration in human colorectal carcinoma [37]. Moreover, the expression levels of these obesity-related genes in cancer tissues were found complex as compared to the normal tissues. The results indicated that the overexpression of these obesity-related genes is associated with the tumor-promoting factors in some specific organs (head and neck, gastrointestinal tract, liver, and gallbladder).

To explore the combined effect of the expression levels of obesity-related genes on the cancer survival rate, the correlation between the expression levels of obesity-related genes and cancer survival rate was assessed by comparing the Kaplan–Meier survival plots among different expression level-based groups. In almost all types of cancer, a significant difference was detected between Kaplan–Meier survival plots of different expression level-based groups. For the majority of obesity-related genes, cancer patients who were in low/medium-expression level group had a superior prognosis than those in the high-expression level group. This finding demonstrated that obesity-related genes may play a critical role in angiogenesis and migration of cancer cells. For example, GPR120 plays a key role in the metastasis of human colorectal carcinoma [37].

However, for three types of cancer (SKCM, ACC, and LUAD), patients in the high-expression group for *GPR120* gene could benefit from a greater prognosis as compared to those in the low/medium-expression level group. Moreover, for four types of cancer (KIRP, UVM, CESC, and LUSC), patients in the high-expression level group for *SH2B1* gene experienced a better prognosis than those in the low/medium-expression level group. These findings indicated that the overexpression of a number of obesity-related genes plays a critical role in the treatment of specific types of cancers. For instance, adipocytes were utilized to regenerate red blood cells in leukemia model [18]. Adipose tissue controls breast cancer progression with the impact of obesity and diabetes [38].

To further investigate the role of obesity-related genes in carcinogenesis, the changes in the expression levels of these genes in carcinogenesis were compared. According to the Kaplan–Meier survival curves, patients with kidney cancer in the low/medium-expression level group for each of *LEPR* and *NEGR1* genes had a long-time life expectancy in comparison to those in the high-expression level group. However, patients with kidney cancer in the high-expression level group for each of *TMEM18* and *SH2B1* genes had a long life expectancy than those who in the low/medium-expression level group. Hence, some obesity-related genes (*LEPR*, *NEGR1*, *TMEM18*, and *SH2B1*) may play critical roles in preventing progression and metastasis of kidney cancer.

To explain the changes in the expression levels of obesity-related genes, numerous molecular processes, such as methylation, mutation, and CNV, for obesity-related genes in cancer tissues and normal tissues were compared. The levels of DNA methylation for obesity-related genes in almost all types of cancer (*HNSC*, *SARC*, *KIRP*, *LUSC*, *PRAD*, *BLCA*, *KIRC*, *LUAD*, *READ*, *BRCA*, *LIHC*, and *COAD*) were altered. Compared to the normal tissues, five obesity-related genes (*POMC*, *LEP*, *PCSK1*, *MTCH2*, and *GPR120*) did show a similarity alteration of DNA methylation patterns in different cancer tissues. Also, no significant differences were observed in the mutation and CNV rates of obesity-related genes between cancer and normal tissues. Therefore, the above-mentioned outcomes suggested that the alterations in DNA methylation patterns could result in the changes in expression levels of obesity-related genes, thereby exerting a critical role in tumor progression.

Once mutations or modify alterations occur for obesity gene, microenvironment probably impacts behavior and adaptive evolution of cancer cells. Meanwhile, location and environmental conditions for cancer cells exposed probably also impact the adaptive capacity. Furthermore, a previous study indicated that the incidence of cancer could be influenced by clinical factors, such as gender, BMI, and history of smoking [4]. Hence, these factors, i.e., gender, race, menopausal status, history of smoking, tumor grade, body weight, and history of drinking, that could impact microenvironment of cancer cells were analyzed. In the present study, Kaplan–Meier analysis was used to assess the combined effect of expression levels of obesity-related genes and the aforementioned clinical factors. The gender and expression levels of obesity-related genes might influence the survival of patients with two types of cancer (HNSC and ESCA). Race and expression levels of obesity-related genes may have a coupled significant effect on the survival of patients with six types of cancer (SKCM, LUSC, TGCT, HNSC, DLBC, and THYM). The menopausal status may remarkably impact the prognosis of BRCA patients. The history of smoking and drinking may influence the survival of patients with LUSC and PAAD. Tumor grade influenced the survival of patients with all types of cancer.

The expression levels of obesity-related genes in cancer tissues and normal tissues revealed the importance of top 25 over/underexpressed genes in the occurrence of cancer. Two obesity-related genes, such as *LEP* and *NEGR1*, may play a substantial role in the occurrence of two types of cancers (BRCA and BLCA). The related genes of obesity (*ADIPOQ*, *GCG*, *PCSK1N*, *TFAP2A*, and *PYY*) were also found to play significant roles in the occurrence of some types of cancer (BRCA, COAD, and UCEC).

**Figure 6:**
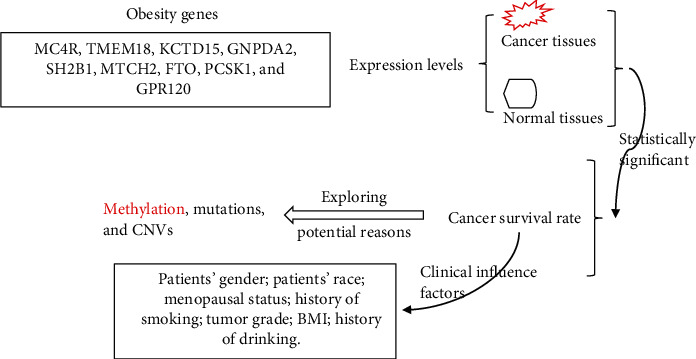
The route line for entire work. Alterations of DNA methylation pattern could result in the changes of expression levels of obesity-related genes.

In conclusion, a significant difference was detected in the expression levels of obesity-related genes between cancer tissues and normal tissues in the current study (Figure 6). For instance, the overexpression of the nine obesity-related genes (*MC4R*, *TMEM18*, *KCTD15*, *GNPDA2*, *SH2B1*, *MTCH2*, *FTO*, *PCSK1*, and *GPR120*) may associate with tumor-promoting factors in some organs. Moreover, the changes in the expression levels of obesity-related genes might influence the survival of patients with different types of cancer (Figure 6). The comparison of changes in the expression levels of obesity-related genes between the occurrence of cancer and patients' survival revealed four obesity-related genes (*LEPR*, *NEGR1*, *TMEM18*, and *SH2B1*), which might play critical roles in preventing the progression and metastasis of kidney cancer. The alterations of DNA methylation patterns could be utilized to explain the changes in expression levels of obesity-related genes (Figure 6). Furthermore, the cancer survival rate was based on the combined effect of the clinical factors and expression levels of obesity-related genes (Figure 6). According to the top 25 over/underexpressed genes for each type of cancer, *LEP* and *NEGR1* were found to be extremely important in the occurrence of BRCA and BLCA cancer. These results provided novel insights into the development of treatment approaches for cancer. However, the mechanism of most obesity genes in tumor progression is still unknown. Meanwhile, molecular functions of most obesity genes are still not fully understood. Hence, a number of in vivo/in vitro experiments would be performed in our future work, to further explore mechanism details of our findings in this study.

## Figures and Tables

**Table 1:** Distribution of obesity genes and their related genes in top 25 over/underexpressed genes.

Query protein	Associated functional protein	Over/underexpressed gene-X^∗^
LEP	SOCS3, STAT3, JAK2, IRS1, PTPN1, INS, LEPR, PPARG, GCG, ADIPOQ	BRCA: LEP-1, ADIPOQ-9 COAD: GCG-18
LEPR	IL6, JAK2, PTPN1, SOCS3, STAT3, PRL, IL10, IL2, LEP, PRKAA1	BRCA: LEP-1
PCSK1	REN, CTSE, GCG, INS, GIP, CTSD, POMC, NAPSA, CPE, PCSK1N	THCA: PCSK1N-21 COAD: GCG-18
MC4R	MC1R, CRHR1, POMC, GLP1R, SDC3, AGRP, CRH, GCG, NPS, IAPP	COAD: GCG-18
TMEM18	GNPDA2, SH2B1, FTO, NEGR1, FAIM2, MTCH2, SEC16B, MC4R, KTCD15, BCDIN3D	BLCA: NEGR1-11
KCTD15	TMEM18, NEGR1, SH2B1, GNPDA2, SEC16B, TFAP2B, TFAP2C, TFAP2A, TFAP2E, TFAP2D	UCEC: TFAP2A-2 BLCA: NEGR1-11
NEGR1	O3FAR1, SDC3, MTCH2, PYY, GNPDA2, SIGLEC15, IGLL5, KCTD15, TMEM18, SH2B1	BLCA: NEGR1-11 COAD: PYY-3
GPR120/O3FAR1	GPR84, MC3R, SLC6A14, FFAR3, PCSK1, GCG, FFAR2, FFAR1, GPR1, GNAT3	COAD: GCG-18

^∗^The order of over (blue)/underexpressed (red) genes.

## Data Availability

The original data used to support the findings of this study are available from the corresponding author upon a reasonable request.

## Supplementary Materials

Supplementary Materials The supplementary material file consists of two Supplementary Tables. Table S1: top 25 over/underexpressed genes for each of 16 types of cancer. Table S2: the distribution of obesity genes and their related genes in top 25 over/underexpressed gene.
