# Supplementary material for: A Combined Effect of Expression Levels of Obesity-Related Genes and Clinical Factors on Cancer Survival Rate
Source: Biomed Res Int. 2020 Nov 24;2020:8838676. doi: 10.1155/2020/8838676 (PMC7707943; doi:10.1155/2020/8838676)
Supplement: Supplementary Materials — The supplementary material file consists of two Supplementary Tables. Table S1: top 25 over/underexpressed genes for each of 16 types of cancer. Table S2: the distribution of obesity genes and their related genes in top 25 over/underexpressed gene. [file 8838676.f1.docx]

**A combined effect of expression levels of obesity-related genes and clinical factors on cancer survival rate**

Ting Huang^1*^, Xuan Huang^2*^, Yumin Nie^3^, Xiangkui Shi^1#^, Chuanjun Shu^3#^

^1^Department of Pharmacy, Xuzhou Maternity and Child Health Care Hospital, Xuzhou, 221000, China.

^2^Reproductive Medical Center, Jinling Hospital Affiliated to The Medical School of Nanjing University, Nanjing, 210002, China.

^3^Department of Bioinformatics, School of Biomedical Engineering and Informatics, Nanjing Medical University, Nanjing, 211166, China.

^*^Contributed equally.

^#^Corresponding information: Xiangkui Shi, jsxzsxk@126.com; Chuanjun Shu, [chuanjunshu@njmu.edu.cn](mailto:chuanjunshu@njmu.edu.cn).

Table S1. Top 25 over/under-expressed genes for each of 16 types of cancer

Table S2. The distribution of obesity genes and their related genes in Top 25 Over/Under-expressed gene

| Query protein | Functional protein association networks | Over/Under-expressed gene X* |
| --- | --- | --- |
| POMC | 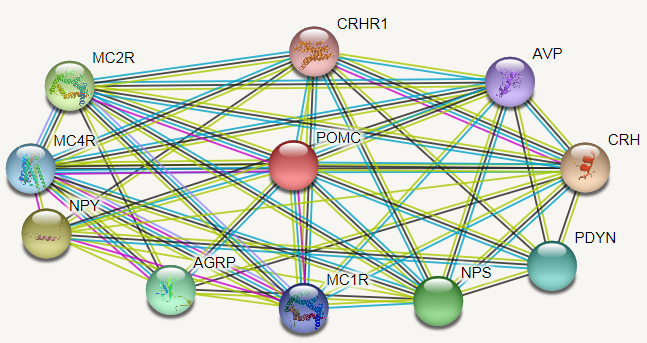 | NA |
| GNPDA2 | 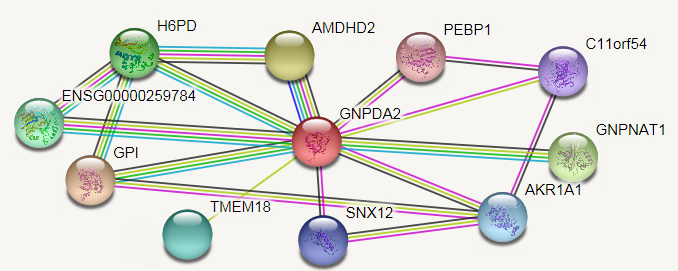 |  |
| SH2B1 | 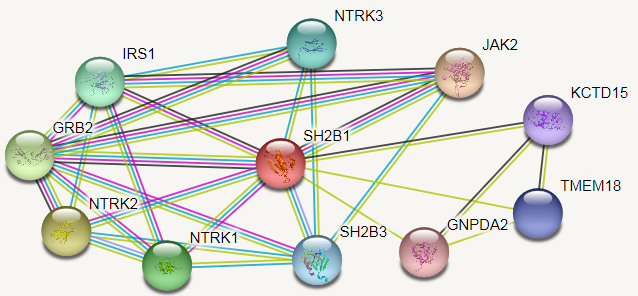 |  |
| MTCH2 | 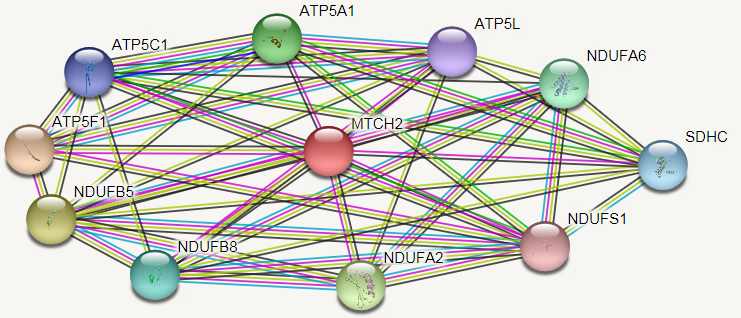 |  |
| FTO | 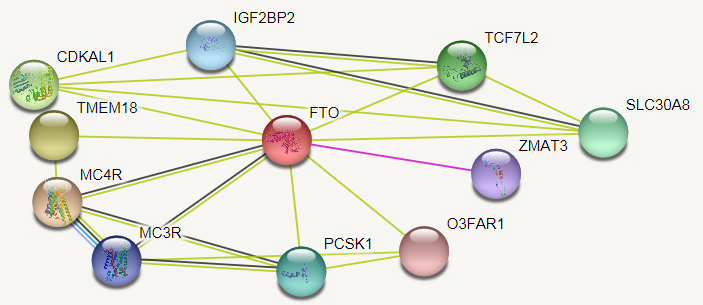 |  |
